# Initial-Stage Suicide Bereavement Experiences: A Case Study

**DOI:** 10.1177/00302228221095905

**Published:** 2022-04-23

**Authors:** Yan Chen, Aarno Laitila

**Affiliations:** 1 Department of Psychology, 4168 University of Jyväskylä, Jyvaskyla, Finland

**Keywords:** suicide bereavement, initial stage, case study, Assimilation Analysis, Assimilation Model

## Abstract

This study aimed to shed light on the initial-stage bereavement experiences of an individual bereaved by suicide, at three months from the loss of his spouse to suicide. A semi-structured in-depth interview was conducted with the individual, a man in his thirties. The data were analyzed using qualitative assimilation analysis, based on the Assimilation Model and the Assimilation of Problematic Experiences Scale (APES). The APES ratings of the interview revealed that the individual’s bereavement was associated with the earlier stages of APES (all scoring under 3.5). In addition, the swift and frequent fluctuations in the APES ratings gave indications that the bereavement was unstable and complicated. It is suggested that mental health professionals could use APES to evaluate suicide bereavement and take note of the APES evaluations in clinical interventions.

## Introduction

Surviving the death of a family member is challenging, especially when the death is due to suicide. Shneidman (1973) claimed that in cases of suicides the greatest public health issue is mitigation of the impact of the loss on those bereaved. Estimations of the numbers of persons bereaved per suicidal death have varied from six (Shneidman, 1972) to ten (Wrobleski, 1991), while the latest estimate of those exposed per suicide has risen to 135 (Cerel et al., 2019). It should be noted that among those left behind by suicide, there is significant variation in the aftermath of the loss, since it encompasses those exposed to suicide, those affected by suicide, and those bereaved by suicide (Andriessen, 2009; Andriessen & Krysinska, 2012; Berman, 2011; Cerel et al., 2014; Crosby & Sacks, 2002). The present study focused on the subset of persons most greatly impacted by suicide, namely those bereaved by suicide. Note that in this paper several synonymous terms are used: “persons bereaved by suicide,” “the suicide bereaved,” and the simplified forms “those bereaved” and “the bereaved.”

The need to study individuals’ lived experiences of suicide bereavement is based on the impact of the loss (Chakraborty & Halder, 2018; Spillane et al., 2017), the large number of those bereaved (Cerel et al., 2019), and the risk of additional suicides and of various mental health disorders among the bereaved (Runeson & Åsberg, 2003; Zhang et al., 2005). According to the framework proposed by Jordan and McIntosh (2011), bereavement following suicide shares some common characteristics with bereavement after all types of loss, including elements of bereavement after unexpected deaths and violent deaths. However, beyond these shared reactions, suicide bereavement has various qualitatively unique and complex characteristics that distinguish it from bereavement following nonsuicidal deaths (Jordan, 2001). These will be summarized below.

Initially, shock, accompanied by numbness and disbelief, may occur due to the unexpected nature of the suicide (Andriessen & Krysinska, 2012), although those who have experienced an intensive “suicide watch” may feel a sense of relief (Sveen & Walby, 2008). In addition, persons bereaved by suicide tend to experience a number of negative feelings towards themselves; these can include heightened feelings of guilt, self-blame, and perceived responsibility for the loss (Bailley et al., 1999; Chapple et al., 2015; Kõlves et al., 2019). At the same time, they may also feel adverse emotions emanating from the loved one, involving feelings of rejection (Chakraborty & Halder, 2018) and a sense of desertion by the deceased (Andriessen & Krysinska, 2012). This can generate intense anger toward the deceased, as well as deep feelings of unworthiness regarding oneself (Chakraborty & Halder, 2018; Jordan, 2008; Kõlves et al., 2019). A unique element of suicide bereavement is a search for answers, and a pondering on unanswered questions, mainly consisting of reasons for the suicidal death, and involving sense-making and meaning-making regarding the death (Castelli Dransart, 2013; 2017; Cerel et al., 2013). This may very well lead to dramatic changes in one’s belief system, encompassing life, the self, others, and the world (Bell et al., 2012; Janoff-Bulman, 1992).

Suicidally bereaved individuals are faced with a higher possibility of suffering from mental problems, and may go through an extremely long and complicated bereavement process (Cerel et al., 2013; Cvinar, 2005; Peters et al., 2016). The manifestations consist of a variety of mental difficulties and disorders (de Groot et al., 2006; Hibberd et al., 2010; Jordan, 2008; Mckinnon & Mckinnon, 2014; Nam, 2016). In addition, grieving family members may also encounter negative experiences in their social network, including stigma, shame, embarrassment, withdrawal, avoidance, loneliness, and isolation (Bell et al., 2012).

At the same time, the surviving families are also likely to experience a series of changes at the family level. Disenfranchised grief may restrict openness in communication and in perception within the family (Bell et al., 2012). This may progress to distortion or closure of communication, and to occurrences of family secrecy (Cerel et al., 2008; Nelson & Frantz, 1996). The guilt, anger, and blame that family members feel toward each other and toward themselves can contribute to silence, maintained in order to cover potentially terrible accusations (Lukas & Seiden, 2007). Some dysfunctional families in which suicide has occurred can experience the same or even a higher risk of dysfunction following their suicidal loss (Jordan, 2001). Moreover, suicide itself has the possibility to distort family patterns, and develop dysfunctional family dynamics (Jordan, 2001).

Findings on the distinctive features of suicide bereavement, as experienced by individuals and families, have focused on the bereavement process (Gaffney & Hannigan, 2010), certain aspects of suicide bereavement (Castelli Dransart, 2013; Cvinar, 2005; Mitchell et al., 2004; Nam, 2016; Talseth & Gilje, 2017; Wood et al., 2012), and the lived experiences of suicide bereavement (Begley & Quayle, 2007). However, in these studies there has been little focus on suicide bereavement experiences at the *initial* stage, though these have been touched on in several other studies (Kõlves et al., 2019; Mitchell et al., 2004; Ross et al., 2018). In addition, no systematic, methodologically robust tools have been applied to characterize the complicated inner world of the suicide bereaved at the initial stage of their bereavement. An understanding of the concrete features of the initial stage of suicide bereavement could assist professionals in providing tailored help to the bereaved. To achieve this aim, in this study we chose *qualitative assimilation analysis* (Stiles et al., 2004; Tikkanen et al., 2013; Tikkanen & Leiman, 2014) as a tool for data analysis, since it has proved to offer particular advantages for microanalytic research (Stiles et al., 1990).

Assimilation analysis is based on the Assimilation Model (AM) and the Assimilation of Problematic Experiences Scale (APES). AM was first utilized to study the changes occurring within psychotherapies (Honos-Webb et al., 1998; Osatuke & Stiles, 2006; Penttinen et al., 2017). Subsequently, the scope of AM developed to new contexts, including gathering data from non-therapeutic interviews (Henry et al., 2009; Moore et al., 2014; Osatuke et al., 2011). The present study represents a further extension of AM, i.e., towards suicide bereavement. The data were drawn from a non-therapeutic interview, the aim being to demonstrate an individual’s lived experiences of suicide bereavement at the initial stage.

The AM method considers problematic experiences as separate, active voices within the person. The self is seen as consisting of a community of voices (Mair, 1977). The voices are formed from traces of associated experiences, and are connected by meaning bridges (Honos-Webb & Stiles, 1998). The community smoothly assimilates voices of unproblematic experience; by contrast, the self may avoid voices composed of problematic experiences, leaving them to form nondominant voices (Brinegar et al., 2006; Honos-Webb et al., 2003). The self becomes stronger and more intact as it incorporates more of a person’s problematic experience.

Assimilation involves the building of a meaning bridge that combines an unaccepted, nondominant voice with an established self/community of voices, which is represented by a dominant voice (Honos-Webb et al., 1999). The sequence of the assimilation levels (APES) reflects a varying association between a dominant voice and a nondominant voice (Honos-Webb & Stiles, 1998). Two basic entities—*topic* and *theme*—are included in the AM. A topic refers to an expressed attitude toward an object (which can be a person, thing, event, or situation), whereas a theme is defined as an attitude revealed recurrently, possibly regarding several objects (Stiles et al., 1991).

In the process of assimilation, the community of voices accommodates the problematic voices through a process that can be divided into eight predictable stages, from stage 0 to stage 7. These can be summarized in terms of the Assimilation of Problematic Experiences Scale (APES) (Stiles et al., 2004). The eight-stage process includes both *cognitive* and *affective* features. At stage 0, the problematic voices are denied or avoided; the affect may be minimal (Stiles et al., 2004). At stage 1, the preference is for problematic voices not to be mentioned; hence they are suppressed or avoided, coming to light only when stimulated by external circumstances, and accompanied by strongly unpleasant but intermittent affect. At stage 2, the problematic voices enter prolonged awareness, but without formulation of the problem, and with acutely painful affect. At stage 3, the problems are clarified, and the opposing voices are distinguished; the affect is unpleasant but exists within the individual’s control, not in a state of panic. It is only at stage 4 that understandings between the separate voices are reached, with problematic experiences being formulated and understood to some extent, along with mixed affect encompassing negative and positive experiences. At stage 5, the understandings are applied to solve problems, while the voices collaborate to find solutions to problems in daily living, with pleasant and optimistic affect. At stage 6, the voices can be utilized with flexibility; the affective tone is pleasant and satisfied. At stage 7, the voices are completely assimilated, growing into resources for dealing with new situations, accompanied by positive or neutral affect.

In our study, AM made it possible to identify particular problematic experiences related to a family member’s suicide. Wilson (2011) used AM to evaluate bereavement counseling. Differing somewhat from his research, our study focused on the use of assimilation analysis to analyze specifically suicide bereavement, in the context of non-therapeutic research interviews.

In seeking to underpin our qualitative research approach, we specifically applied a case study approach as having the capacity to delineate real-life situations and substantial details (Flyvbjerg, 2006). This is in line with most published research on AM (Basto et al., 2018; Laitila & Aaltonen, 1998; Osatuke et al., 2011; Penttinen et al., 2017). AM has been used as a tool for qualitative research, especially when a case study approach has been applied to theory-building (McLeod, 2010; Stiles, 2007). In the present study we used a single case involving only one person who was bereaved by suicide, recruited in China (People’s Republic of China). Our aims centered around the following questions:

What is the nature of suicide bereavement experiences at the initial stage?

How is AM applicable to the analysis of suicide bereavement experiences?

## Method

### Participant

This case study formed part of a larger research project concentrating on the suicide bereavement experiences of persons bereaved in China. Participant W (a pseudonym) had lost his wife to suicide three months prior to the interview. In manifesting the shortest time interval after suicide, W was unique in the entire data corpus within the project. He was interviewed four times, i.e., at around 3 months, 7 months, 10 months, and 18 months from the loss. The first interview is included within this article, since it fulfilled the goal of clarifying suicide bereavement experiences at the initial stage (which has scarcely been studied previously). In addition, W’s first interview was adequately informative. His ways of expressing the situation as he experienced it were vivid and detailed, and the richness of his descriptions made the interview a good basis for an intensive case study—always bearing in mind the need for sensitivity and for a strictly ethical approach (see below). In terms of personal background W was a man in his thirties. He had received a higher education. He had been married for 4–5 years. The marriage was the first for both W and his late wife, and they did not have children.

### Research Ethics

The fieldwork of the research project commenced after ethical approval was obtained from the affiliated university’s ethics committee. Suicide is such a sensitive topic that ethical issues were clearly of paramount importance in research of this kind. Before the interview, the first author—who was also the interviewer, and who is a Chinese female clinical psychology doctoral student and certified psychological counselor—introduced to W the purpose and procedures of the interview. She mentioned both the potential benefit and risks pertaining to the research, with an emphasis on the voluntary and anonymous nature of the participation. She informed W of his right to withdraw from the interview at any time, and the resources available if he experienced negative emotions aroused by the interview. Questions raised by W were answered. Immediately before the interview began, a written informed consent form was signed.

Great emphasis was placed on caring for W’s feelings and well-being during the entire interview, and also for his mental well-being after the interview. The stance of the interviewer, and the process of the interview, were greatly influenced by the belief that researchers must learn from the suicide bereaved and enter the field with a “not-knowing” attitude (see Dyregrov, 2011). Throughout the interview, the interviewer mostly followed W’s focus on his bereavement experiences, giving him the initiative and freedom to decide what to express. In this way it was intended that the interviewee could gain more control regarding the autonomous management of his own emotions, and of the pace of the narration during the interview. After the interview, the interviewer undertook follow-up inquiries on the participant’s mental well-being so that support could be offered when needed.

### Procedures

#### Entering the Field—Recruitment of the Participant

W was recruited through a suicide bereavement support group in an economically developed city in China. First of all, the interviewer contacted and met the group leader, an experienced psychologist working in a psychiatric hospital. After discussion of the research project and hearing the opinions of the group members, it was agreed that the interviewer could take part in the group meetings on several occasions, as a volunteer and as a researcher. The group had regular monthly meetings in which the interviewer participated on two occasions. It was during these that the interviewer got to know W. During the interaction between the interviewer and W within the group meetings, W was willing to openly talk about his bereavement experiences with the interviewer, and he showed great interest in the research. After discussing W’s emotional stability with the group leader and getting an affirmative answer, the interviewer invited W to participate in the study.

#### Data Collection—Interview

A face-to-face, semi-structured in-depth interview was conducted with W. The interview focused on his bereavement experiences and his bereavement process, and further on his emotional reactions, perception, changes, and ways of coping at different times. The interview occurred in a quiet and private venue, with the aim of making W feel safe and uninterrupted. The 144-minute interview was recorded and subsequently transcribed verbatim.

#### Analysis of the Interview

The first author conducted the interview in China. She subsequently transcribed the interview from an audio recording to a verbatim transcript, and translated the transcript into English. Initially, she conducted the assimilation analysis through listening to the audio recording, and through reading the Chinese transcript. The audio recording was a good basis for perception of W’s emotions, as prosodic features such as volume, tone, pauses, sighing, and trembling in the voice could be vividly heard, and hence taken into account in the analysis. Meanwhile, the second author, a Finnish psychologist with extensive experience in clinical psychology research, went through the translated transcript and conducted the assimilation analysis. After the independent and concurrent assimilation analysis conducted by the first and second author, the two authors performed collaborative data analysis within regular data sessions (face-to-face meetings and online video meetings). The analytical procedure used was adapted from a four-step assimilation analysis previously used to analyze psychotherapy sessions (Brinegar et al., 2006; Stiles & Angus, 2001). At every step, the independent data analyses alternated with collaborative data sessions. Each step was iterative until consensus was achieved.

Step 1 Familiarization and Cataloguing. Through listening to the audio recording and reading the Chinese transcript (with the second author reading the translated transcript), W’s thoughts and feelings regarding his wife’s suicide were noted, and a list was made of the problematic topics.

Step 2 Identifying Problematic Voices and the Community of Voices. From the list of topics extracted in Step 1, one central theme, namely W’s wife’s suicide, was identified. Within this theme, seven voices, including four dominant voices and three nondominant voices, were distinguished on the basis of their content and emotion.

Step 3 Excerpting Passages. Passages representing the seven voices were located and excerpted. After this step, 21 passages representing the voices were selected.

Step 4 Describing the Process of Assimilation Represented in the Passages. APES ratings were assigned to each of the 21 passages screened in Step 3, and the reasoning for the ratings was clarified. The development of specific voices was noted, as were interactions and conflicts between voices.

## Results

### Overview: W’s Community of Voices and Nondominant Voices

**Figure 1.**
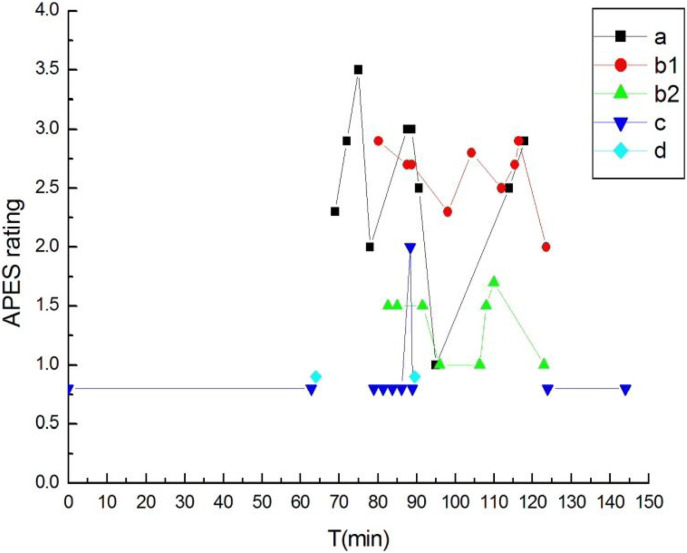
Occurrence of Voices, and Changes in the APES Ratings over the Entire Interview.

The main product of the four-step data analysis is summarized in Table 1 below. We identified one central theme from the interview, i.e., *W’s wife’s suicide*. There were two pairs of conflicting voices, as listed in Table 1. These consisted of (1) the *self-regulation* voice as opposed to *uncontrollable emotions,* and (2) *normalcy of life* as opposed to *accidental death/suicidal death*. The occurrence and the changes in the APES ratings of the voices are presented in Figure 1. In the following sections we shall elaborate how these voices developed in the interview.

**Table 1.** Voices within W’s Self.

Reference by letters *a–d* (see Figure 1)	Community of voices	Problematic voices
***c***	Rationalizing	
***d***	Self-observation	
***a***	Self-regulation	Uncontrollable emotions
***b***	Normalcy of life	Accidental death (b1)
		Suicidal death (b2)

The *rationalizing* voice was manifested when W wandered among various scientific and philosophical topics accompanied by negative but comparatively calm emotions (APES 0.8). From Figure 1 it can be observed that the *rationalizing* voice (represented by line *c* in Figure 1) was the only voice present in the first 63 min after the interview began, and in the last 21 min before the end of the interview, with intermittent occurrences also from 79:03 to 90:50 min, where it was accompanied by other voices. With the *rationalizing* voice, W seemed to involve himself in rational thinking, and to temporarily avoid talking about his wife’s suicide. Within this phase, W was mostly close to the stage of “active avoidance,” i.e., APES 0.8. However, one exception appears at 88:24, when the APES rating of the rationalizing voice reaches 2.0. The excerpt below presents one example of W’s *rationalizing* voice.

Excerpt 1: (61:16–61:36)

W: Then, in fact, from junior high school, I began to have an interest in philosophy though I didn’t know it was philosophy back then, but after I read it, I felt it rather suited my preference......

The emergence of W’s *self-observation* voice implied less avoidance than before (hence representing APES 0.9). From around the 63^rd^ minute, with the *self-observation* voice, W’s narratives moved in such a way as to be more internally related to himself, as compared to the external topics taken up previously.

Excerpt 2: (63:09–65:14)

W: Mostly, I feel like I am searching for myself in others.

Int: Do you find yourself in someone else?

W: Sometimes after others do stupid things, everyone may laugh at the person, but I don’t laugh. I know that although I haven’t done it to the extent he did, I have some ideas in my mind that are the same as his......

Throughout the interview, the interaction within the two pairs of conflicting voices, and the interweaving between them, constituted an important part of W’s assimilation of the suicide. We shall present these voices in the following sections.

### Conflicting Pairs of Voices

#### Self-regulation Voice versus Uncontrollable Emotions Voice

The dominant *self-regulation* voice caused W to regulate his emotions so that his relatively normal life could be sustained. The nondominant *uncontrollable emotions* voice opposed the *self-regulation* voice; it represented the expression of emotions which were hard to control. From Figure 1 one can see that the two voices were manifested in the middle section of the interview; here, the APES ratings of this pair of voices (represented by line *a*) went as low as APES 1.0 and as high as APES 3.5. Otherwise, most of the ratings were situated between APES 2.0 and APES 3.0. 10 excerpts containing the *self-regulation* voice and the *uncontrollable emotions* voice were extracted for APES ratings. Due to space limitations, we shall report on only two of these in this article.

Excerpt 3: (68:04–70:28)

Int: (Silent for 4s) What do you think has been the biggest impact on you of the thing that happened in your family?

W: (Silent for 8s) Grief (pause for 8s, drinks water, long inhalation). My wife (voice trembling, pause for 2s) is/was (in Chinese, “is” and “was” are expressed by the same Chinese word) not a very mature person mentally, sometimes (voice trembling)......after this happened (pause for 4s), I don’t know how to say it, perhaps I can’t say anything out of rationality, but from the point of view of the emotions (pause for 5s), from the emotions, that is a very strong feeling (pause for 4s), out of rationality (pause for 4s), out of rationality, for me, from a rational point of view, there’s nothing to say, the thing is she is gone ...

W did not mention his wife’s suicide for the first 68 min of the entire interview. At the 68 min, seizing the chance presented by four seconds of silence, the interviewer referred to his loss in a relatively implicit manner (“the thing”), seeking attunement with the interviewee. From his reply, two voices could be extracted: *self-regulation* and *uncontrollable emotions*. He appeared to focus on *self-regulation*, indicating only once his *uncontrollable emotions* (briefly, after which he went on to revert to *self-regulation*). These two voices appeared differentiated and separate, and there was not yet any dialogue between them. Meanwhile, in the recording, W’s tone of voice was sad, trembling, and suggestive of strong emotion; it contained frequent pauses, implying a struggle to control his intense emotional pain. Based on these manifestations, we assessed his APES score as 2.4 at this point, i.e., as located between vague awareness/emergence and problem statement/clarification.

Excerpt 4: (74:45–76:30)

W: Talking with people, talking with people, to see if it is possible to let this feeling out, maybe this is the way the mutual aid group works, this thing is not like what Freud…, even Freud couldn’t do anything to make it all..., I can only find an opportunity to let this feeling out, but you can’t remove all the feelings and make it so there’s nothing left. It’s very powerful, continuous, and endless, and then of enormous strength.

Int: Umm

W: I regard it as an existence. This kind of existence makes me a little uncomfortable, not so comfortable. I know it is normal (after the thing happened), but I know this thing will happen, I think, if I want to know myself, know, how to, not about getting rid of it or overcoming... Closer to how to be a friend with this feeling.

In this passage, the dialogue between the s*elf-regulation voice* and the *uncontrollable emotions* voice achieved APES 3.5, i.e., at the mid-point between (stage 3) problem statement/clarification and (stage 4) understanding/insight. The *uncontrollable emotions* voice was clarified. The two voices were approaching the point of working together to have problems resolved, but that point had not yet been reached. The overall situation of his emotions was still negative, but more manageable than before.

#### Normalcy of Life Voice versus Accidental Death/Suicidal Death Voice

The *normalcy of life* voice represented W’s ideology about what life was supposed to be normally; by contrast, the *accidental death/suicidal death* voice represented his perception of his wife’s death. W’s perception of his wife’s death was somewhat contradictory. On the one hand, he conceived his wife’s death as an unexpected accident due to a misadventure in wrongly taking medicine (see excerpt 5). On the other hand, he took part in both the bereavement support group and the interview, and in the latter, he mentioned the “depressed” state, the “low mood”, and the poor well-being status of his wife. In addition, after his wife died, he spent a lot of time looking up information on suicide and depression. These behaviors suggested that he might not be denying the possibility that his wife died from suicide. Beyond the *normalcy of life* voice, it seemed that W’s perception regarding his wife’s death could be divided into two branches—the *accidental death* voice and the *suicidal death* voice. These two branches sometimes merged into each other, while mostly existing separately. The three voices were present in the middle part of the recorded interview. In general, the APES ratings on the relationship between *normalcy of life* and *accidental death* were higher than those exhibited between *normalcy of life* and *suicidal death*, as can be seen from Figure 1 (lines b1 and b2). The former branch was rated between 2.0 and 2.9, while the latter was rated between 1.0 and 1.7. In total, 16 excerpts containing the *accidental death* voice and the *suicidal death* voice could be extracted for APES ratings. Below, we report on one excerpt, within which only three voices (*normalcy of life*, *accidental death*, and *suicidal death*) were present. For clarity, the non-dominant voices and the APES ratings are given in parentheses after the corresponding sentences.

Excerpt 5: (93:10–110:20)

Int: What do you think of her choosing this action?

W: I don’t think it was her choice (suicidal death: 1.0). She took some medicine that shouldn’t be taken (pause for 12s)......At that time, she was not here. She went to her parents’ home. When the thing (accidental death: 2.3) happened, they happened to have given her those pills to take around those few days......I know this must not have been her choice...... (she) went home for a while and (they) told me that she was gone, I didn’t believe it at all.

Int: She was not very well before the thing happened, right?

W: (She) couldn’t fall asleep, had pain in her body, then (they) let her take the tablets. Those tablets were for her father. The precautions said severe depression can lead to aggravated sickness and a suicidal tendency. My wife was in a low mood back then. I kept describing a good future to her......This is how the diary was written seven days before her departure (pause for 9s) (accidental death: 2.7), that’s why I didn’t believe that she chose to [xxx] (inaudible) herself. (suicidal death: 1.0).

Int: Why was she unhappy during that time? What happened?

W: She had a pain in her body......this sickness happened to her (confused tone), how much was it a blow to her? It was never so serious that she would commit suicide (pause for 13s) (suicidal death: 1.0)...... She was on leave from work. It was good that her family could stay with her 24 hours a day, nothing would happen, this was a very reassuring thing (suicidal death: 1.5).

Int: Taking too much of the tablets will lead to depression?

W: People who are depressed can’t take the tablets (tone raised). ......my wife, like a little girl, not a strong person, otherwise she wouldn’t have felt so upset and nagged all day long after getting this disease (clearing throat) (she) is/was not a strong person, she was the youngest, she was spoiled when she was young (suicidal death: 1.7).

### Interweaving of Voices and Swift Shifts in APES Ratings

**Figure 2.**
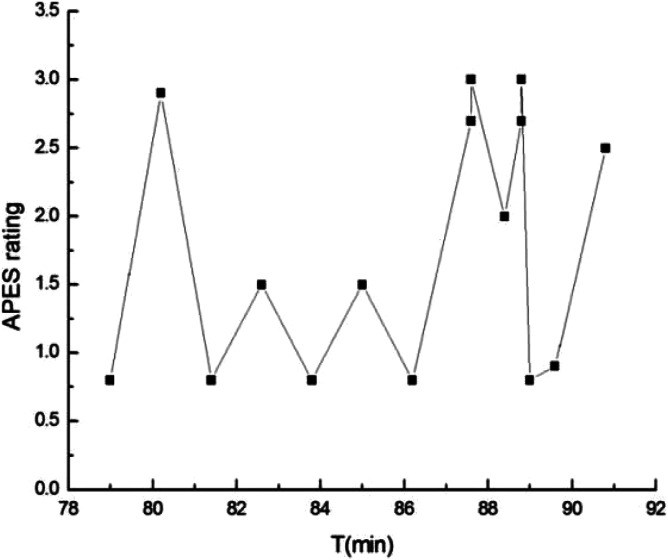
Interweaving of Voices, and Rapid Shifts in APES Ratings.

There was one section of the interview in which all the seven voices were present. Here, frequent swift shifts in the APES ratings appeared within a period of around 10 min. Unlike the excerpts presented above, the voices in this passage did not appear on their own; on the contrary, they were interwoven and mixed with each other. Moreover, higher ratings of the voices appeared only sporadically, and were frequently interrupted by other voices with lower APES ratings (see Figure 2 below).

Excerpt 6: (79:03–90:50)

W: Life is like a game or a farce...... more often there are no rules......We consider ourselves the center of the universe, in fact we are just one......(rationalizing: 0.8) when I saw my wife’s bone ashes (accidental death: 2.9), I thought about this, one day I will also be like this (choking with sobs)......That’s why I like reading some philosophical stuff......psychology......Vipassana......philosophy......(rationalizing: 0.8) then you will find the human brain is a rational self. These kinds of things actually have rationality in the backstage. But I don’t think like this about depression (suicidal death: 1.5), I am 99% sure it is caused by endocrine dyscrasia. Everyone’s endocrine system is different...... (rationalizing: 0.8). I believe that if there is this kind of irrational and irresistible feeling of depression, I think it’s at least 95% related to the brain and endocrine system (suicidal death: 1.5).

Int: The human brain is rational?

W: It’s the human brain......when you play games, your hands shake like this. What your hands feel is not something purely psychological, psychology is actually part of the body, the spirit and the body are actually unitary...... Dualism is a simplification......Psychology is too complicated......how can you actually use your own consciousness to study yourself. I think the human brain is an automated machine, the backstage has a lot of programs, it is almost impossible for you to use the front stage to study the backstage (rationalizing: 0.8). Sometimes you can feel you experienced what someone else is going through now, the power of experience...... my goodness, on that day, the feeling of powerlessness I felt was that I was tiny (accidental death: 2.7/uncontrollable emotions: 3.0), and suddenly I understood why foreigners have religious beliefs and Chinese people do not have religious beliefs .....I read one book...... (rationalizing: 2.0). At that moment, I felt that mood...... (accidental death: 2.7/uncontrollable emotions: 3.0). Those Greek deities (rationalizing: 0.8)......I think actually my life was pretty smooth..., so many years, no massive blow or anything... (self-observation: 0.9).

Int: Um, (pause for 5s), but this thing is a massive blow right?

W: I don’t know the starting point of your question. It must be a massive blow to my private life, but it has no impact on my career, for my family and life, it is a...... The impact on the emotions is relatively large (uncontrollable emotions: 2.5).

Within these (approximately) 10 min, as exhibited by the APES ratings, regardless of which voice the ratings belonged to there were alternations of relatively higher ratings and lower ratings. In Figure 1, from time point 79:03 to 90:50, we connected all the rating points in chronological order, and found the fluctuations to exhibit a sawtooth pattern (see Figure 2).

## Discussion

The present study aimed to use AM to illustrate W’s initial stage of bereavement, at three months from the loss of his spouse to suicide. In addition, we attempted to explore AM’s applicability to analyzing suicide bereavement experiences. Finally, we anticipated that we would be able to enrich the research on the suicide bereavement processes occurring at the initial stage.

Our assimilation analysis of the interview with W indicated that the initial stage of suicide bereavement was complicated and unstable. The seven voices within W’s self could be rated in terms of different APES stages. The voices exhibited differences in how they intertwined and related to each other, with frequent and swift shifts between ratings in the part of the interview analyzed. These features all contributed to the overall picture of complexity and instability in W’s internal process of assimilating his loss. Another aspect that made W’s bereavement both complicated and distinctive was W’s contradictory perception concerning his wife’s death, veering between the two voices of “accidental death” and “suicidal death”. Interestingly, at some points, the two branches of W’s perception regarding his wife’s death seemed not to be adversarial, in the sense that there was no clear borderline between them. The higher assimilation of the “accidental death” voice might imply that on the whole, W would have preferred to perceive his wife’s death as accidental; in this way his psychological suffering would have been reduced.

We gave APES ratings to each of W’s dominant voices and to each of his conflicting pairs of voices; however, we did not assess W’s overall APES rating in assimilating his wife’s suicidal death. The complexity and the frequent fluctuation between the APES ratings of the seven voices made it hard to give a clear-cut overall APES rating. Despite this, it was possible to draw a conclusion from Figure 1, namely that W’s overall assimilation of the suicide was still at an early stage in APES terms. This could indicate that W was at an initial point in his bereavement journey, and that he still had a considerable way to go before fully assimilating his suicidal loss.

The fluctuation was manifested more dramatically when swift shifts appeared between the APES ratings of all the seven voices in the course of around 10 min, creating a sawtooth pattern. This pattern has been observed in several previous studies (Osatuke et al., 2005; Penttinen & Wahlström, 2013; Stiles & Angus, 2001). However, the previous studies found the pattern in the context of psychotherapies, and the pattern consisted of APES ratings from different psychotherapy sessions—unlike the present study in which the pattern was exhibited within a period of around 10 min in a single non-therapeutic interview. The sawtooth pattern illustrated how W mourned his wife’s suicide, following a natural course, without receiving professional support, or any help other than from the bereavement support group. During the (approximately) 10 min, W’s utterances on his wife’s death alternated with distancing topics. The alternation between topics could be his own conscious or unconscious self-soothing strategy. Coupled with his avoidance of mentioning his loss in the first 68 min of the interview, one can observe that W was initially reluctant to talk about grief. The study of Chan and Cheung (2020) on suicide bereaved Chinese men found a similar phenomenon. There it was explained as a normal response to loss which could help them to address their hidden bereavement.

The second aim of this research was to explore how AM is applicable in analyzing suicide bereavement experiences. By illustrating the seven voices and their APES stages, plus their dynamic interaction, the application of AM made the complexity and nuances of W’s bereavement with accompanying multi-dimensionality of emotions visible and specific. Compared to other qualitative analysis methods (Begley & Quayle, 2007; Gaffney & Hannigan, 2010; Ross et al., 2018), we suggest that AM is able to make visible not only the theme of the narratives, but also the hidden voices underneath the theme. In such a case, AM appears to allow a clearer and more in-depth understanding of the complex inner process of suicide bereavement experiences.

From the perspective of suicide bereavement at the initial stage, we noted the complexity and instability in W’s suicide bereavement, in line with several previous studies on suicide bereavement (Cerel et al., 2013; Cvinar, 2005; Peters et al., 2016). Similarly, Ross et al. (2018) found adaptation to suicide bereavement to follow a dynamic and shifting course at six months and 12 months after the loss. However, since the present study included one single case, further verification is needed as to whether frequent fluctuation in APES ratings is a feature unique to the initial stage of suicide bereavement. We anticipate that relevant comparisons will be made in subsequent analyses within the current research project.

Our analysis revealed that W showed certain distinctive features in processing his suicidal loss. Indeed, every individual who has been bereaved through suicide may have qualitatively distinct paths through bereavement (Hall, 2014). W’s alternation in narration between various scientific/philosophical topics and his loss-related experiences could have formed his own efficient way of maintaining a balance between his restless emotions and his ongoing life demands. In addition, one can see that at some points W’s emotions were detached from self-regulation, which was dominated by his rationality. In this regard, Gaffney and Hannigan (2010) explained the detachment of emotions at the initial stage of bereavement as acting as a self-defensive strategy, minimizing the traumatic impact of the suicidal loss on the bereaved person. The fact of the loss may initially be so overpowering that bereaved persons need to become detached in order to manage their everyday functioning (Gaffney & Hannigan, 2010).

### Strengths and Limitations

For the sake of credibility of the study, the research setting, the participants, and the data analysis have been presented here through “thick” description, giving as much detail as space allows (Creswell & Miller, 2000). Furthermore, close collaboration between the two authors was seen as providing a further guarantee of the credibility of the data analysis.

Nevertheless, one must be aware that the possibilities of generalizing from this study are limited, given that it involves one single case exhibiting (one may assume) distinct characteristics as regards bereavement experiences. Moreover, because of the low accessibility of suicide bereaved individuals in China, sampling was based on convenience, meaning that we cannot exclude the possibilities of selection bias. Another limitation is that our data do not allow us to make any valid cultural interpretations regarding suicide bereavement. Some cultural features may indeed have been present in the interview, but our research procedures involved a focus rather on the private and individual processes of suicide bereavement.

Despite the limitations above, it can be claimed that our single case study illustrated the research participant’s initial stage of bereavement experiences in depth and in detail. It provides a unique portrait of the internal process of adapting to suicidal loss, and a comprehensive overview of the dynamic interaction between the voices within the process. The combination of AM with suicide bereavement research made the nuances of the internal processes visible. Hence, our study seems well placed to extend the (so far) limited knowledge on the bereavement experiences of the suicide bereaved, and especially knowledge on the initial stage of suicide bereavement, bearing in mind that very few previous studies have focused on this phase.

It can be claimed that the researcher-participant relationship in this study exhibits both strengths and limitations. The interviewer took part in the bereavement support group’s regular meetings on two occasions. This appeared to leave W enough time to consider his participation; in addition, familiarity with the interviewer before the interview was favorable in creating a sense of safety such that W could volunteer to participate. This helped to build rapport, allowing the interviewer access to crucial narratives in W’s experiences, with richer and more authentic data. However, in such a case, the interviewer and interviewee might well develop unconscious preconceived opinions about each other. Hence, W’s interview could well have had features different from those carried out with people outside the group, in terms of the interview process and the content of the data.

### Clinical Implications

On the basis of this study, it can be claimed that health professionals could usefully apply forms of assimilation analysis to gain a clear portrait of the internal process of adapting to suicidal loss, with possibilities for more specific guidance on the intervention. Moreover, as indicated by this and by other studies, professionals may bear in mind that instability, complexity, and (very possibly) ambivalence can accompany the bereaved through their initial bereavement. Helping bereaved persons to cope with instability, complexity, and ambivalence merits a strong initial emphasis in clinical intervention, and can be expected to form one of the main standards in assessing the psychosocial support provided at this stage.

## Conclusion

The application of AM shed light on initial suicide bereavement experiences. In terms of AM, the research participant was still at the initial stage of his bereavement process, and had a considerable way to go before fully assimilating his suicidal loss. Professionals should aim to establish a rapport with bereaved persons that will allow them to consider their distinct characteristics, and by applying assimilation analysis professionals may gain a clearer understanding of the inner conflicts of the bereaved. Knowledge of suicide bereavement at the initial stage (and specifically, suicidal loss at three months post-death) can contribute to determining the most appropriate ways to alleviate the negative impacts of the complicated and unstable psychological states experienced, with possibilities for improving the mental health status of bereaved family members.
